# Spatiotemporal expression profiling of long intervening noncoding RNAs in *Caenorhabditis elegans*

**DOI:** 10.1038/s41598-017-05427-5

**Published:** 2017-07-12

**Authors:** Weihong Liu, Enchao Yu, Siyu Chen, Xiaopeng Ma, Yiwen Lu, Xiao Liu

**Affiliations:** 1 MOE Key Laboratory of Bioinformatics, Center for Synthetic and Systems Biology, School of Life Sciences, Tsinghua University, Beijing, 100084 China; 2 PTN (Peking University-Tsinghua University-National Institute of Biological Sciences) Joint Graduate Program, Beijing, 100084 China; 3 Department of Automation, Tsinghua University, Beijing, 100084 China

## Abstract

To better understand the biological function of long noncoding RNAs, it is critical to determine their spatiotemporal expression patterns. We generated transgenic reporter strains for 149 out of the 170 annotated *C. elegans* long intervening noncoding RNAs (lincRNAs) and profiled their temporal activity. For the 68 lincRNAs with integrated reporter lines, we profiled their expression at the resolution of single cells in L1 larvae, and revealed that the expression of lincRNAs is more specific, heterogeneous and at lower level than transcription factors (TFs). These expression patterns can be largely attributed to transcriptional regulation because they were observed in assays using reporters of promoter activity. The spatial expression patterns of the 68 lincRNAs were further examined in 18 tissue categories throughout eight developmental stages. We compared the expression dynamics of lincRNAs, miRNAs and TFs during development. lincRNA and miRNA promoters are less active at embryo stage than those of TFs, but become comparable to TFs after embryogenesis. Finally, the lincRNA gene set shows a similar tissue distribution to that of miRNAs and TFs. We also generated a database, CELE, for the storage and retrieval of lincRNA reporter expression patterns and other relevant information. The data and strains described here will provide a valuable guide and resource for future functional exploration of *C. elegans* lincRNAs.

## Introduction

An increasing number of long noncoding RNAs have been identified across species. However, most of them are uncharacterized and of unknown function. Recent efforts have begun to explore the function of long noncoding RNAs. In vertebrates, a set of long noncoding RNAs were reported to have multiple functions in development and cell differentiation, and were involved in a wide range of molecular processes such as transcriptional regulation, epigenetic regulation, sequestration of microRNAs, splicing, protein translation and stability^1–5^. In addition, abnormal expression of long noncoding RNAs has been shown to lead to development defects or diseases^1, 6–8^. In invertebrates, functional studies of long noncoding RNAs are also underway. In *Drosophila*, the long noncoding RNA CRG regulates locomotor behavior^9^; *yar* affects sleep behavior^10^; *acal* functions in epithelial shape changes during dorsal closure^11^. In *C. elegans*, the function of only a few long noncoding RNAs has been reported. *tts-1* has high expression level in life-extending *daf-2* and *clk-1* mutants, and it is required for lifespan extension^12^. Another long noncoding RNA, *rncs-1*, whose transcription is induced by starvation, can modulate expression of Dicer-regulated genes^13^.

Long noncoding RNAs have poor evolutionary sequence conservation and the predicting the function of the majority of long noncoding RNAs is difficult^14^. So far, the most commonly used method to categorize long noncoding RNAs is according to their position relative to neighboring genes^15^. In *C. elegans*, Nam and Bartel identified 170 long intervening ncRNAs (lincRNAs) that did not overlap protein-coding transcripts, and 58 antisense long noncoding RNAs that were complementary to protein-coding transcripts. In addition, they found that long noncoding RNAs tended to express in a tissue- or stage-specific manner^16^. Similarly, many long noncoding RNAs are reported to exhibit highly tissue- or cell type-specific expression pattern in mammals and flies^17–20^. Here, we focus on the 170 annotated lincRNAs which have presumed independent promoters, and profile their spatiotemporal expression patterns. *C. elegans* is especially suitable to study spatiotemporal gene expression because of the essentially invariant cell lineage, transparent body and convenience of gene transformation with a visible fluorescent protein. Here, we use an automatic cell lineage analyzer to generate expression data of lincRNA reporters in 363 cells of L1 larvae at the resolution of single cells^21^.

Though terminal cell fates such as neuronal, pharyngeal and intestinal fates have been established in L1 larvae, some precursor cells continue to divide and differentiate during post-embryonic development. For example, P neuroblasts divide several times and give rise to ventral cord motor neurons, ventral hypodermis and vulva; seam progenitor cells undergo a stem-cell-like division and contribute an additional 98 nuclei to the hyp7 syncytium; and two founder cells Z1 and Z4 that are present in the L1 gonad primordium generate all cells of the somatic gonad. The expression pattern of a single stage is insufficient to reveal the dynamics of gene expression. Therefore, we further profiled our lincRNA reporters in all developmental stages.

In this work, we generated 260 transgenic *C. elegans* strains with reporters for 149 lincRNAs and measured their expression patterns. For the 68 integrated lincRNA reporters, we profiled their expression in 363 somatic cells of L1 stage larvae at single-cell resolution. These data revealed that the spatiotemporal distribution of lincRNAs is similar to that of TFs, but lincRNA reporter expression is more tissue specific and heterogeneous. Furthermore, lincRNA and miRNA promoters are less active than those of TFs in embryos. For convenient storage and retrieval of lincRNA expression patterns and other related information, we also generated a database, *C.elegans* lincRNA expression (CELE, wano.bioinfo.org). The results and reagent generated in our study will provide a valuable resource to explore the biological function of *C. elegans* lincRNAs.

## Results

### Characterization of PlincRNA::reporter transgenes

We generated reporter constructs for each annotated lincRNA by inserting its 5′ regulatory region into a GFP or mCherry expression vector (PlincRNA::reporter). The promoter sequences were defined as the intergenic sequences upstream each lincRNA’s annotated first exon, ranging from 301 bp to 6505 bp (Supplementary Table S1). The cloned promoters of 82% (130/157) of lincRNA genes covered their whole intergenic upstream sequence. Five lincRNA genes (*linc-32*, *linc-57*, *linc-95*, *linc-110*, *linc-136*) have long first introns, indicating they contain some cis-elements. Therefore, the reporter constructs of these five genes include their first introns as well. The intergenic upstream regions of the rest genes (14%, 22/157) are too long to clone. In these cases, at least 2 kb of nematode-conserved intergenic sequences were used as the promoter. It has been demonstrated that promoter sequences defined by these criteria can accurately recapitulate expression patterns of endogenous protein-coding and miRNA genes^22–24^. The expression vector also contains the coding region of histone H1 fused to the reporter gene, which will produce a stable nuclear localized fluorescent protein. Transgenic *C. elegans* strains carrying the PlincRNA::reporter construct were generated by microparticle bombardment as previously described^25^.

In total, we generated 260 transgenic *C. elegans* strains covering 149 lincRNAs. Fluorescence was detectable in 95% (142 out of 149) of the PlincRNA::reporter transgenes (Supplementary Fig. S1a; Supplementary Table S1). The expression rate of PlincRNA::reporters is comparable to that of miRNAs (90%) and TFs (92%)^22, 23^. Seven lincRNA promoters failed to drive detectable fluorescent protein expression, including *linc-36*, *linc-86*, *linc-101*, *linc-127, linc-129*, *linc-133*, and *linc-135*. The RNA expression level of *linc-86, linc-133* and *linc-135* is low in the modENCODE RNA-seq dataset, while *linc-127* has higher expression in males than in hermaphrodites^26^. The cloned promoters of *linc-36*, *linc-101*, *linc-129* and *linc-135* are shorter than 2 kb, so may lack cis-elements required for expression.

We examined the expression of the 142 lincRNAs whose promoter reporters have detectable activity throughout all developmental stages: three embryo stages (early, middle and late), four larval stages (L1–L4) and adult. To compare our reporter data to previously generated RNA-seq data, we digitized lincRNA temporal expression patterns from the modENCODE RNA-seq data (Supplementary Table S2 and Methods Section)^26^. We found that temporal expression patterns of 60% (86 out of 142) of our PlincRNA::reporters are consistent with those detected by RNA-seq (Supplementary Fig. S1b; Supplementary Table S2). The extent of consistency between the PlincRNA::reporters and the RNA-seq data is similar to the degree of consistency between the temporal expression pattern of PmiRNA::reporters and Northern blotting data (65%)^22^. In another words, the temporal expression patterns of our reporters are largely consistent with the developmental expression patterns seen by RNA-seq.

**Figure 1:**
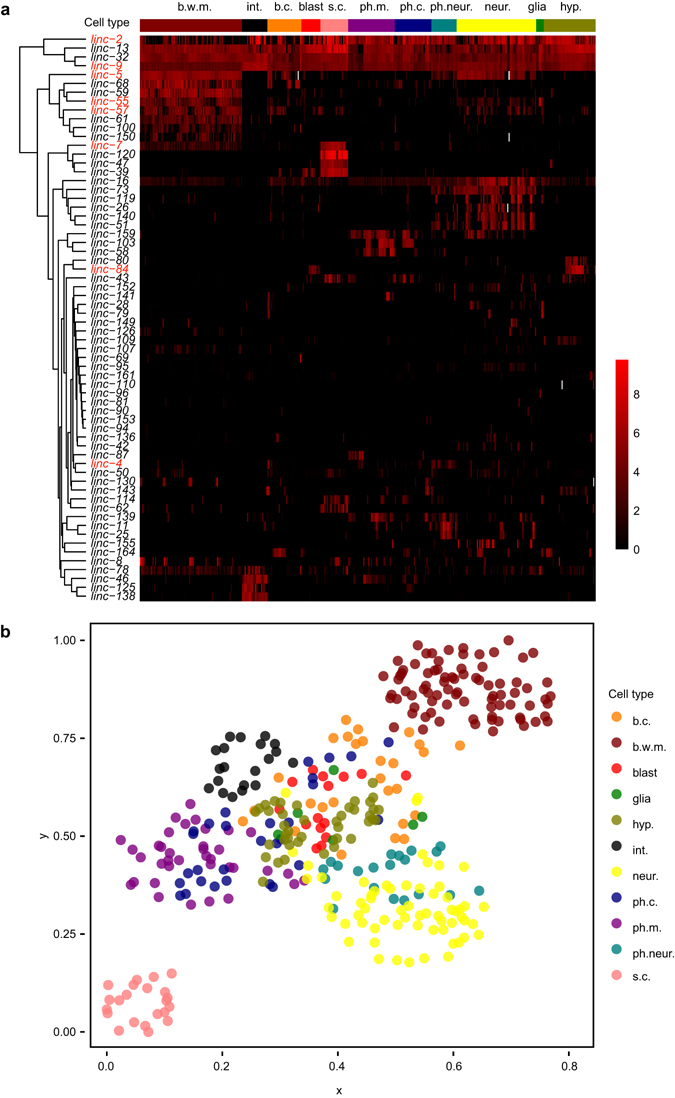
The single-cell expression profiles of lincRNA reporters. (**a**) lincRNA reporter expression profiles in newly hatched L1 larvae. The expression level of 64 PlincRNA::reporters in 361 somatic nuclei is shown. The gene expression level is adjusted by calculating (gene expression level + 500)/500, and is plotted as the log2 (adjusted gene expression level). Genes (rows) were clustered according to their expression pattern. 361 somatic cells (columns) were manually arranged according to their cell types and cell position along the anterior-posterior axis of the worm. Grey entries in the heatmap represent missing cells. The data used to generate this figure can be found in Supplementary Table S3. Gene names of eight lincRNAs that are significantly expressed in muscle according to previous SRT-based RNA-seq are in red^27^. (**b**) 361 somatic cells clustered by lincRNA promoter reporter expression. A terrain map of nuclei was generated by Genesis according to correlation of gene expression between cells^35^. Each dot represents a nucleus. Colors indicate different cell types. The distance between two nuclei in the x-y plane indicates similarity in gene expression. Cells with similar gene expression patterns are close to each other, while cells with different patterns are far apart. b.c., other body cells; b.w.m., body wall muscle; blast, blast cells; glia, glia cells; hyp., hypodermal cells; int., intestine cells; neur., neurons; ph.c., other pharyngeal cells; ph.m., pharyngeal muscle; ph.neur., pharyngeal neurons; s.c., seam cells.

Next, we compared the tissue specificity of lincRNAs detected by our reporter assay by RNA-seq. A trans-splicing-based RNA tagging (SRT) approach has been used for muscle-specific RNA-seq in *C. elegans* ^27^. This RNA-seq data covered 40 lincRNAs whose promoter reporters were included in our L1 stage profiling, and eight of these 40 lincRNAs were significantly expressed in muscle according to their RNA-seq data^27^. Seven of these eight genes showed significant expression in muscle cells in our PlincRNA::histone1::reporter transgenic worms (Fig. 1a), further suggesting that reporter activities are largely representative of the expression patterns of their endogenous genes. However, several factors may cause the deviation of reporter expression patterns from endogenous ones. Some important cis-elements may not be included in cloned promoters. Additionally, posttranscriptional processing may affect expression but would not be measured by a reporter of promoter activity^22^. Lastly, lincRNAs may be activated only in specific conditions. For example, *linc-3* is specifically expressed in intestinal cells of dauer worms^16^ and the transcription of long noncoding RNA *rncs-1* is induced only by starvation^13^. Furthermore, some lincRNAs are reported to be male-specific^26^, but we only examined reporter expression in hermaphrodites.

### Quantitative single-cell gene expression profiling of L1 larvae

We profiled the expression of 64 out of the 68 integrated lincRNA reporters in newly hatched L1 larvae using an image analysis pipeline that annotates 64% (363 out of 558) nuclei of L1 larvae, except those closely arranged head neurons (Supplementary Table S3)^21^. Four lincRNAs were not included, *linc-3*, *linc-89*, *linc-93* and *linc-160*. *linc-3* is exclusively expressed in intestine at dauer stage (Supplementary Fig. S2), consistent with previously reported RNA-seq data^16^. *linc-89* and *linc-93* are expressed in unidentified head neurons in L1 larvae (Supplementary Table S4). *linc-160* is expressed in a few unidentified somatic gonad cells only from L2 to L4 stages (Supplementary Tables S2,S4). We converted the quantitative expression values into a heatmap showing a broad overview of lincRNA expression patterns (Fig. 1a). Only about 5% of lincRNA genes (*linc-9*, *linc-13*, *linc-32*) are expressed ubiquitously, significantly fewer than TFs reported in previous study (25%, 15 out of 59)^21^. This result indicates that lincRNAs are expressed in a more restricted manner than TFs. Some lincRNAs have tissue-specific expression patterns, such as *linc-120*, *linc-47* and *linc-39* in seam cells, and *linc-5*, *linc-68* and *linc-59* in body wall muscle. Other lincRNAs such as *linc-8* and *linc-130* are specifically expressed in head body wall muscle, but not in trunk or tail. The biological significance and regulatory mechanism of such heterogeneous expression within a tissue remains unknown. To test whether lincRNAs and their neighboring genes tend to be co-expressed, we compared the expression patterns of tissue specific lincRNA reporters and their adjacent upstream and downstream protein coding genes in the genome. In our L1 stage profile, there are 23 tissue-encriched lincRNA genes whose upstream and downstream protein-coding genes have reported expression patterns, but there was no significant correlation between their expression patterns (Supplementary Table S5).

To evaluate the reproducibility of PlincRNA::reporter expression patterns, we examined multiple transgenic lines for 17 genes (Supplementary Table S6). The correlation coefficient of gene expression patterns between transgenic lines of the same reporter constructs (R = 0.72) is significantly higher than that of different reporter constructs (P-value < 0.001) (Supplementary Fig. S3), and comparable to that of 12 protein coding gene reporters in a previous publication using same imaging-based method as this study (R = 0.80)^21^. We examined genomic features such as promoter length, position relative to neighboring gene, gene size and the number of exons of these 17 genes (Supplementary Table S7). None of these features significantly correlated with the reproducibility of gene expression patterns. We further clustered 361 cells into groups in a two-dimensional scatter plot according to their correlation in lincRNA gene expression. As expected, cells of the same type tended to cluster together (Fig. 1b).

### Comparison of the expression patterns of lincRNA and TF reporters

**Figure 2:**
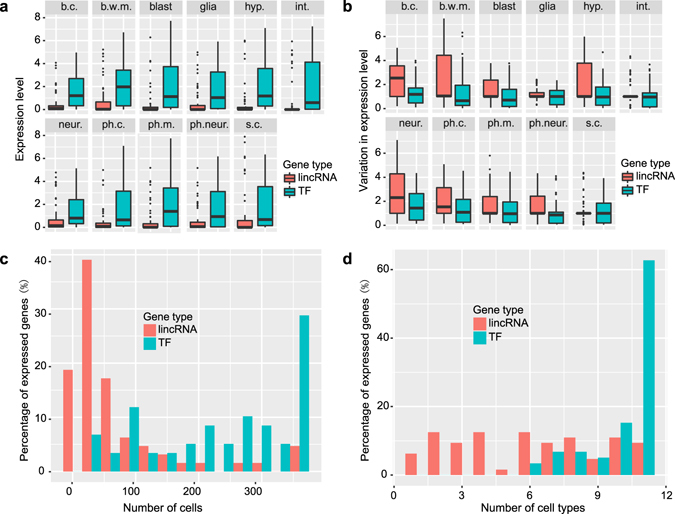
Comparison of expression features of lincRNA and TF reporters. (**a**,**b**) Expression level (**a**) and the coefficient of variation in expression level (**b**) of lincRNA and TF reporters in each cell type (CV is calculated as the the ratio of the standard deviation σ to the mean μ). Cell type abbreviation is the same as in Fig. 1. Error bars equal SEM. (**c**,**d**) Distribution of the number of cells (**c**) and cell types (**d**) in which lincRNA or TF reporters are expressed.

Several studies have reported that of lincRNAs have lower expression levels and exhibit more tissue-specific or stage-specific patterns than protein coding genes in various organisms^17, 28–30^. Here, we compare the expression patterns of 64 lincRNA reporters and 59 TF reporters in *C. elegans* L1 larvae^21^. First, we calculated the average gene expression level in 11 different cell types for each gene set. We found that the average expression level of lincRNAs is much lower than that of the TFs in every cell type (Fig. 2a), which is consistent with previous RNA-seq results that the mean RPKM of *C. elegans* lincRNAs is much lower than that of mRNAs^16^. Second, we evaluated the variation in gene expression between cells of the same type and found that the expression of lincRNA reporters is more heterogeneous than that of TFs (Fig. 2b). Finally, we investigated the distribution of lincRNAs and TFs across different cells and cell types. A large proportion of the examined TF reporters are expressed in more than 200 cells, while most lincRNA reporters are expressed in fewer than 100 cells (Fig. 2c). Similarly, more than 60% of TF reporters are expressed in all cell types, while fewer than 10% of lincRNA reporters are expressed in all cell types (Fig. 2d).

In summary, lincRNA reporters not only have lower expression levels and higher variation in gene expression, they also exhibit more cell/tissue- specific expression patterns than TF reporters. Because these results are based on the activity of promoter reporters, these observed gene expression patterns can mostly be attributed to transcriptional regulation.

### Characterization of spatiotemporal lincRNAs gene expression

**Figure 3:**
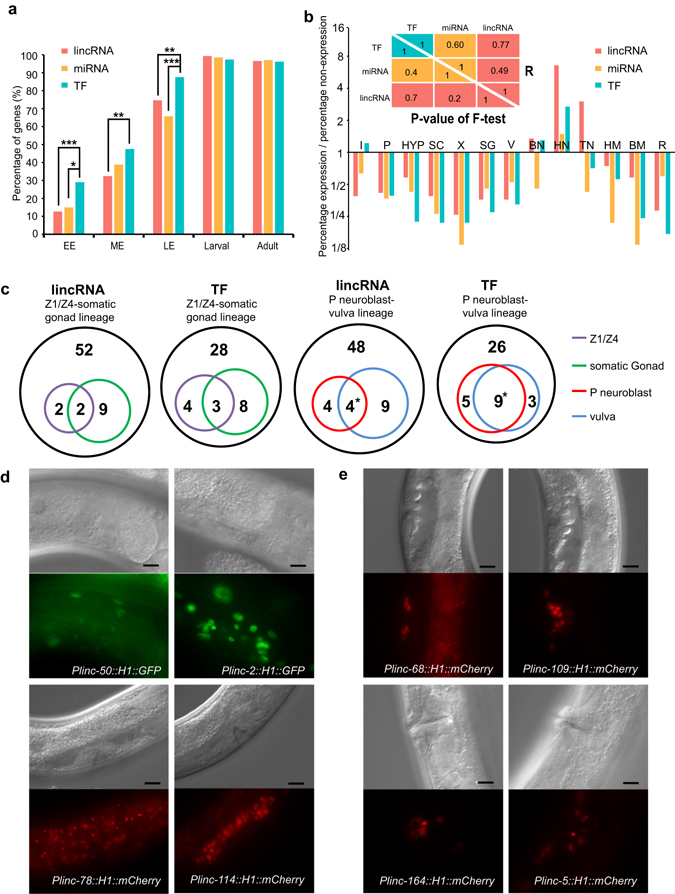
Comparison of spatiotemporal expression patterns of lincRNA, miRNA and TF reporters. (**a**) Temporal expression patterns of lincRNA, miRNA and TF reporters. Genes in each of the three categories whose promoter reporters show detectable activity are included in the comparison (lincRNA, 142; miRNA, 67; TF, 335). EE, early embryo (pre-comma stage); ME, middle embryo (comma to 1.5-fold stage); LE, late embryo (2-fold to 3-fold stage). *P < 0.05; **P < 0.01; ***P < 0.001, Fisher’s exact test. Detailed information can be found in Supplementary Table S2. (**b**) Spatial expression patterns of lincRNA, miRNA and TF reporters. The percentage of each gene set expressed in a tissue is shown as an odds ratio. The upper right half of the triangle table shows the correlation of the probabilities of expressed genes in each somatic tissue between the three gene sets, in which Pearson correlation coefficients were calculated based on the log2(odds ratio); The lower left half of the triangle table shows the non-significant P-values resulting from a test of whether the variance of the probabilities that a gene is expressed in each of the somatic tissues is significantly different between each of the three gene sets, P-values were calculated based on the log2(odds ratio) using an F-test. I, intestine cells and intestinal muscle; P, pharynx; HYP, hypodermal cells; SC, seam cells; X, cells of excretory system; SG, somatic gonad; V, vulva and vulva muscle; BN, ventral nerve cord and body neurons; HN, head neurons; TN, tail neurons; HM, head muscle; BM, body muscle; R, anal depressor muscle, anal sphincter, rectal epithelium and rectal gland cell. Corresponding pictures can be found in the *C.elegans* lincRNA expression (CELE) database (wano.bioinfo.org). Detailed information can be found in Supplementary Table S4. (**c**) Number of lincRNA and TF reporters expressed in the Z1/Z4-somatic gonad lineage and the P neuroblast-vulva lineage. Black outer circle, the number of whole assayed lincRNA reporters and TF reporters. There is a significant overlap between genes expressed in P neuroblasts and in the vulva, P < 0.05, Fisher’s exact test. Detailed information can be found in Supplementary Table S4, S8. (**d**,**e**) Representative lincRNA reporters expressed in somatic gonad (**d**) and in vulva (**e**). Scale bar, 10 μm.

We profiled the temporal expression patterns of 142 lincRNA reporters in in eight developmental stages and the spatial expression patterns of 68 integrated lincRNA reporters in 18 somatic tissue categories. Detailed information and images can be found in Supplementary Tables S2, S4 and the *C. elegans* lincRNA Expression (CELE) database (wano.bioinfo.org).We also re-annotated the previously reported spatiotemporal expression patterns of miRNA and TF reporters to enable a comparison of expression patterns across these three gene sets^22, 23^. We found that lincRNA and miRNA promoters are less active at embryo stage than those of TFs, but become comparable to TFs after embryogenesis (Fig. 3a).

Because lincRNA reporters tend to be more tissue-specific than TFs, we examined whether lincRNA reporter expression is enriched or depleted in specific tissues (Fig. 3b). To control for the effect of subjective tissue classification, we used reporter expression data of TFs and miRNAs as negative controls^22, 23^. First, every somatic tissue expresses some lincRNA, TF and miRNA reporters. Second, the fraction of each gene set expressed in any given somatic tissue is highly correlated between these three gene sets (Fig. 3b, up-right triangle table). Finally, the variance of expressed genes across the somatic tissues is not significantly different between these gene sets (Fig. 3b, lower-left triangle table). In short, lincRNA reporters do not show a more significant bias towards expression in a particular tissue than those of TFs and miRNAs.

A *C. elegans* one-celled zygote gives rise to 558 cell nuclei in newly hatched larvae and 959 somatic cell nuclei in adult hermaphrodite^31–33^. At L1 larval stage, terminal fates such as neuron, pharynx, and intestine have been established. The expression patterns of lincRNA reporters remain largely constant over time in these terminally differentiated tissues (CELE database, wano.bioinfo.org). Therefore, the single-cell expression pattern profiled in L1 larvae is representative of expression in later stages for most tissues.

To demonstrate that the lincRNA promoters are active at examined stages, we performed FRAP (Fluorescence Recovery After Photobleaching) experiments on lincRNA reporters in six cell/tissue types (P*linc-164*::reporter in vulva at L4 stage, P*linc-2*::reporter in somatic gonad at adult stage, P*linc-46*::reporter in intestine at L4 stage, P*linc-5*::reporter in muscle and neurons at L2 stage, and P*linc-120*::reporter in seam cells at L2 stage). We found that the fluorescence signal significantly recovered three hours after bleaching (Supplementary Fig. S4), which is much shorter than the time between adjacent developmental time points examined in this study. This result is largely consistent with modENCODE RNA-seq data, where the expression of four of these five lincRNAs (*linc-2, linc-46, linc-5, linc-*120) were detected at corresponding stages (Supplementary Table S2)^26^. Therefore, our reporter assays provided pertinent information for temporal characterization at the resolution employed in this study.

There are several progenitor cells in L1 larvae that undergo multiple rounds of cell division to generate adult organs or tissues, such as Z1/Z4 that give rise to the somatic gonad and some P neuroblasts that give rise to the vulva. We examined and compared the expression of lincRNA reporters in these progenitor cells and their progenies (Fig. 3c–e, Supplementary Tables S3,S4). We found that there is a significant correlation between which lincRNA genes are expressed in P neuroblasts and in the vulva (P-value < 0.05), similar to terminally differentiated tissues. However, no significant correlation was observed between the genes expressed in Z1/Z4 progenitor cells and those expressed in the somatic gonad. We next asked whether the expression of TFs is also different in Z1/Z4 progenitor cells and the somatic gonad. We examined the expression of 43 TFs in vulva and somatic gonad that had been profiled at L1 stage^21^. Similarly to the lincRNAs, expression of TFs is correlated between P neuroblasts and vulva (P-value < 0.05), but not between Z1/Z4 progenitor cells and the somatic gonad^21^ (Fig. 3c–e, Supplementary Table S8).

### Construction of a *C*. *elegans*lincRNA expression (CELE) database

We constructed a database for the storage and retrieval of our lincRNA reporter expression data called *C*. *e* *legans* lincRNA expression (CELE) database (wano.bioinfo.org). This database also contains additional detailed information including the PCR primers used to clone the promoters, characteristics of the transgenes, and images of reporter expression patterns.

**Figure 4:**
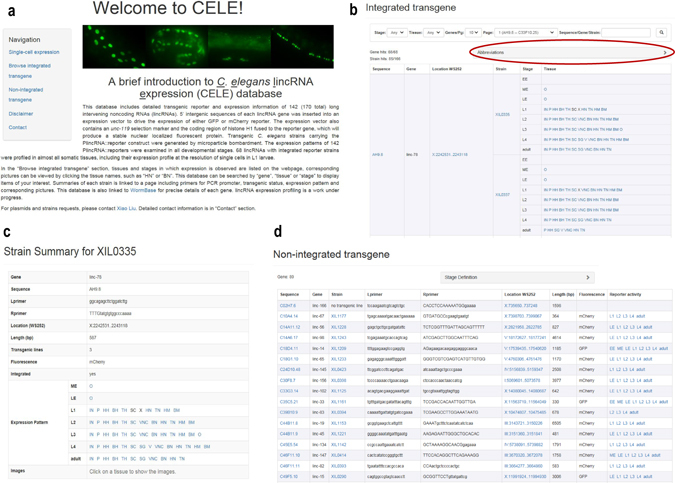
*C. elegans* lincRNA expression (CELE) database. (**a**) CELE database homepage. Hyperlinks at left are for users to navigate to the section of interest. (**b**) “Browse integrated transgene” section. The tissues and stages in which lincRNA reporter expression was observed are listed, and corresponding pictures can be viewed by clicking the tissue names. Definition of the abbreviation for each tissue and stage can be found at the top of the page (red circle). This database can be searched by “gene”, “tissue” or “stage”. (**c**) Strain summary page. (**d**) “Non-integrated transgene” section.

On the left side of the database home page, there are hyperlinks for users to navigate to the section of interest (Fig. 4a). In the “Single-cell expression” section, users can find the heatmap showing the expression of 64 lincRNA reporters in the 361 somatic cells we profiled, and can download a text file containing the quantitative gene expression data used to generate this heatmap. In the “Browse integrated transgene” section, for each lincRNA reporter the tissues and stages in which expression was observed is listed, and corresponding pictures can be viewed by clicking the tissue names. Definition of the tissues and stages can be found at the top of the page (Fig. 4b). The CELE database can be searched by “gene”, “tissue” or “stage”. Both sequence names (*e.g*. T01C8.12) and gene names (*e.g. linc-100*) can be used to search for genes. Gene and promoters are linked to WormBase so that users can easily obtain more information about the gene. The summary of each transgenic strain is linked to a page that contains the PCR primer sequence for its cloned promoter, transgene information, a description of its reporter expression pattern and corresponding pictures (Fig. 4c). In the “Non-integrated transgene” section, representative images of the 81 PlincRNA::reporters that are not integrated into the genome are shown, along with a list of the stages in which each PlincRNA::reporter is expressed. Detailed clone and transgene information is also provided (Fig. 4d). In the “Contact” section, contact information is provided for plasmids or strain requests and user feedback.

## Discussion

In this paper, we present quantitative gene expression data of 64 lincRNAs in 363 somatic cells of L1 larvae. Several lines of evidence indicate that our single-cell expression data is reliable. First, PlincRNA::reporter expression patterns are largely reproducible between transgenic lines carrying the same reporter construct. Second, cells with same fates cluster together based on lincRNA reporter expression. Third, the expression patterns seen in our reporter assay are significantly correlated with previously reported RNA-seq data both spatially and temporally^26, 27^. We compared the expression patterns of 64 lincRNA reporters and 59 TF reporters at single-cell resolution. lincRNA reporters not only have a lower expression level than TFs, they also exhibit greater tissue-specificity. Previous studies in *C. elegans* and other organisms have drawn similar conclusions based on RNA-seq data^16, 29, 30^. However, our results using reporters of promoter activity suggests that transcriptional regulation plays a critical role in lincRNA expression patterns.

We profiled the spatiotemporal expression of 68 integrated lincRNA reporters in 18 tissue categories and eight developmental stages in living worms and compared the expression patterns of lincRNA reporters with those of miRNAs and TFs. lincRNA and miRNA promoters are less active at embryo stage than those of TFs. Although lincRNA expression tends to be more tissue specific than TFs, the pattern of tissues in which lincRNA reporters are expressed is similar to that of TFs. The expression of a lincRNA reporter is almost constant in terminally differentiated tissues during larval development. In addition to terminally differentiated tissues, there are progenitor cells in L1 larvae, such as some P neuroblasts (vulva progenitor cells) and Z1/Z4 (somatic gonad progenitor cells). We found that the expression of both lincRNA reporters and TF reporters is significantly correlated between P neuroblasts and vulva, but no correlation was observed between Z1/Z4 progenitor cells and somatic gonad.

We generated a database for storage and retrieval of single-cell expression datas in L1 larvae, spatiotemporal expression patterns during development, and other relevant information on lincRNAs. Well-established gene expression databases, such as WormBase (http://www.wormbase.org/), WormAtlas (http://gfpweb.aecom.yu.edu/index), EPIC (http://epic.gs.washington.edu/), Hope laboratory expression pattern database (http://bgypc059.leeds.ac.uk/~webuser/) and EDGEdb (http://edgedb.umassmed.edu) have greatly facilitated studies of gene regulation and gene function. The *C. elegans* lincRNA expression (CELE) database will fill the need for a similar database of lincRNA expression patterns. We will continue to add more lincRNA expression data and other resources in the future. We expect that the data on the spatiotemporal expression patterns of lincRNAs will form a foundation for exploring the functions of lincRNA. Furthermore, the availability of expression databases for diverse gene categories will facilitate the exploration of the interactions between proteins and RNAs.

## Methods

### Generation of PlincRNA::reporter constructs

We generated PlincRNA::reporter constructs by inserting the 5′ regulatory regions of each gene of interest into an expression vector pJIM20^34^, which contains an *unc-119* selection marker and a fluorescent protein (GFP or mCherry) fused to the coding region of histone H1. The promoter sequences were defined as the intergenic sequences upstream the each lincRNA’s annotated first exon. Long first introns may contain cis regulatory elements; therefore, we also included the first intron in cases where it was longer than 200 bp. Usually, the whole intergenic upstream sequence should be cloned. However, for genes with intergenic regions greater than 2 kb, the nematode-conserved intergenic sequences of at least 2 kb were used as the promoter. The average length of the promoters that we cloned is 2.1 kb, with a minimum length of 301 bp and a maximum length of 6505 bp. In total, 157 promoters were successfully cloned into the expression vector by conventional restriction-ligation method (Supplementary Table S1).

### Transgenic strain construction

PlincRNA::reporter constructs were introduced into *unc-119* (*ed3*) or *unc-119* (*tm4063*) worms by microparticle bombardment as described previously^25^.

### Generation of single-cell PlincRNA::reporter expression profiles

We generated single-cell PlincRNA::reporter expression profiles consulting the pipeline as previously described^21^, except that cell names were manually annotated according to their position and shape, because crossing these strains with the P*myo-3*::reporter marker strain was impractical for the large number of lines that we generated. In the heatmap, cell names were arranged according to their cell types and genes were clustered according to their expression pattern. Cell type classification information can be seen in Supplementary Table S9. As transgene silencing happens in the germline, two germline progenitor cells, Z2 and Z3, have no detectable reporter expression. Thus, we excluded these two cells in heatmap.

### Characterization of spatiotemporal lincRNAs gene expression

PlincRNA::reporter expression was examined by fluorescence microscopy using a Zeiss Imager.A2 microscope equipped with an AxioCam MRm camera. For expression examination of larval and adult worms, mixed populations of hermaphrodites were mounted on agar pad and were treated with 0.5 mg/ml levamisole. For embryos, a dozen adult worms were picked into a drop of M9 buffer on a coverslip and were cut up to release embryos. Then, embryos were mounted on an agar pad for microscopy. Pictures and detailed PlincRNA::reporter expression information are stored in the *C. elegans* lincRNA expression (CELE) database (wano.bioinfo.org) and Supplementary Tables S2, S4.

### FRAP (Fluorescence Recovery After Photobleaching)

Worms were mounted on agar pad and were treated with 0.5 mg/ml levamisole, and the edge of the coverslip was sealed with Vaseline to prevent the agar pad from drying out too quickly. We took a fluorescence image of a worm before photobleaching, photobleached all the fluorescence in the field, and took a second fluorescence image after photobleaching. We let the worm recover for 3 h at 25 °C, and imaged again using the same exposure time and settings.

### PlincRNA::reporter expression pattern annotation

We recorded the spatiotemporal expression of each PlincRNA::reporter in a standardized table representing reporter expression in binary code (1 means expression detected, 0 means expression undetectable) as previously described^22^. Temporal expression pattern includes eight developmental stages: early embryo (pre-comma stage), middle embryo (comma to 1.5 fold stage), late embryo (2 to 3 fold stage), L1–L4 larval stages and adult stage. Detailed information about tissue classification is shown in Supplementary Table S10. To compare the expression patterns of lincRNA reporters with those of miRNA and TF reporters^22, 23^, we re-arranged the expression pattern of these two data sets and merged our 18 categories into 13 (Supplementary Table S4).

### lincRNA temporal modENCODE RNA-seq data re-annotation

The modENCODE project collected expression data from seven classical developmental stages (early embryo, late embryo, L1–L4 larval stages and adult stage)^26^. We converted the quantitative temporal modENCODE RNA-seq data to a binary code (1 means FRPM > 0, 0 means FRPM = 0) to compare it to our data from the PlincRNA::reporters. If expression is present or absent in both our data and the modENCODE data in five or more developmental stages for a given lincRNA gene, we considered the measurement consistent between these two assays for this gene.

## Electronic supplementary material

SupplementaryMaterials

Dataset 3

Dataset 6

Dataset 1

Dataset 2

Dataset 4

Dataset 5

Dataset 7

Dataset 8

Dataset 9

Dataset 10
