# Supplementary material for: Spatiotemporal expression profiling of long intervening noncoding RNAs in Caenorhabditis elegans
Source: Sci Rep. 2017 Jul 12;7:5195. doi: 10.1038/s41598-017-05427-5 (PMC5507858; doi:10.1038/s41598-017-05427-5)
Supplement: Supplementary file 1 — SupplementaryMaterials [file 41598_2017_5427_MOESM1_ESM.pdf]

Supplementary Materials for

# Spatiotemporal expression profiling of long intervening noncoding RNAs in *Caenorhabditis elegans*

Weihong Liu[1], Enchao Yu[1], Siyu Chen[1], Xiaopeng Ma[1,2], Yiwen Lu[3], Xiao Liu[1*]

[1]MOE Key Laboratory of Bioinformatics, Center for Synthetic and Systems Biology, School of Life Sciences, Tsinghua University, Beijing 100084, China

[2]PTN (Peking University-Tsinghua University-National Institute of Biological Sciences) Joint Graduate Program, Beijing 100084, China

[3]Department of Automation, Tsinghua University, Beijing 100084, China

[*]To whom correspondence should be addressed. Tel: +86-10-62792304; Fax: 86-10-62788604; Email: xiaoliu@tsinghua.edu.cn

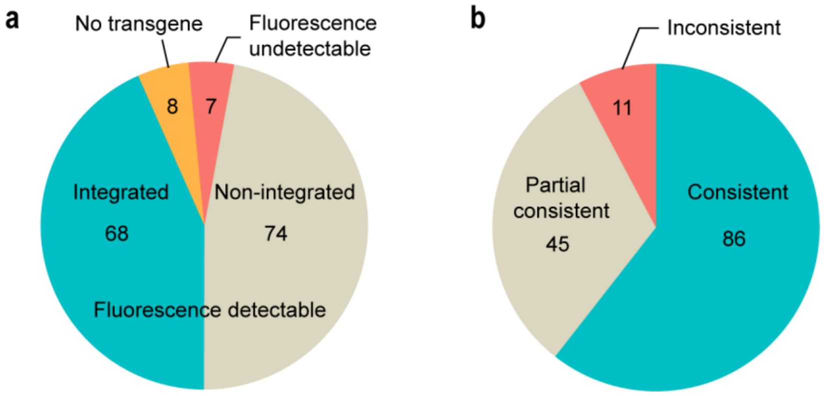


**Supplementary Figure S1. Characterization of PlincRNA::reporter transgenes.** (**a**) Status of PlincRNA::reporter transgenes. (**b**) Chart showing the fraction of PlincRNA::reporters consistent with temporal expression patterns compared to lincRNA expression data from modENCODE RNA-seq.

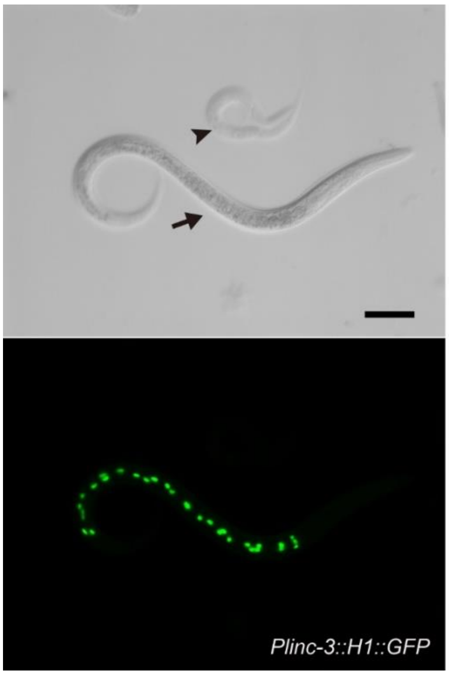


**Supplementary Figure S2. Specific expression of *linc-3* in intestine at dauer stage.** Arrowhead, L1 larva; arrow, dauer stage worm. Scale bar, 50 µm.

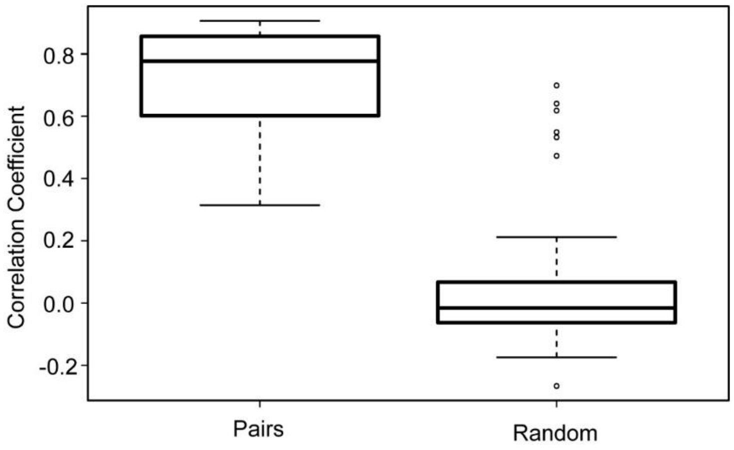


**Supplementary Figure S3. Reproducibility of single-cell gene expression profiles.** A comparison of the expression correlation between worms from different transgenic lines carrying the same reporter construct and worms transformed with different reporter constructs. 17 lincRNAs reporters have two or more transgenic lines used for single-cell gene expression analysis. The Pearson correlation coefficients of single-cell gene expression patterns between different transgenic lines with the same reporter construct (Pairs) and 100 randomly sampled pairs (Random) from the 82 transgenic strains used for single-cell gene expression profiling were calculated. Error bars, SEM. P = 1.519e-14, Welch Two Sample t-test.

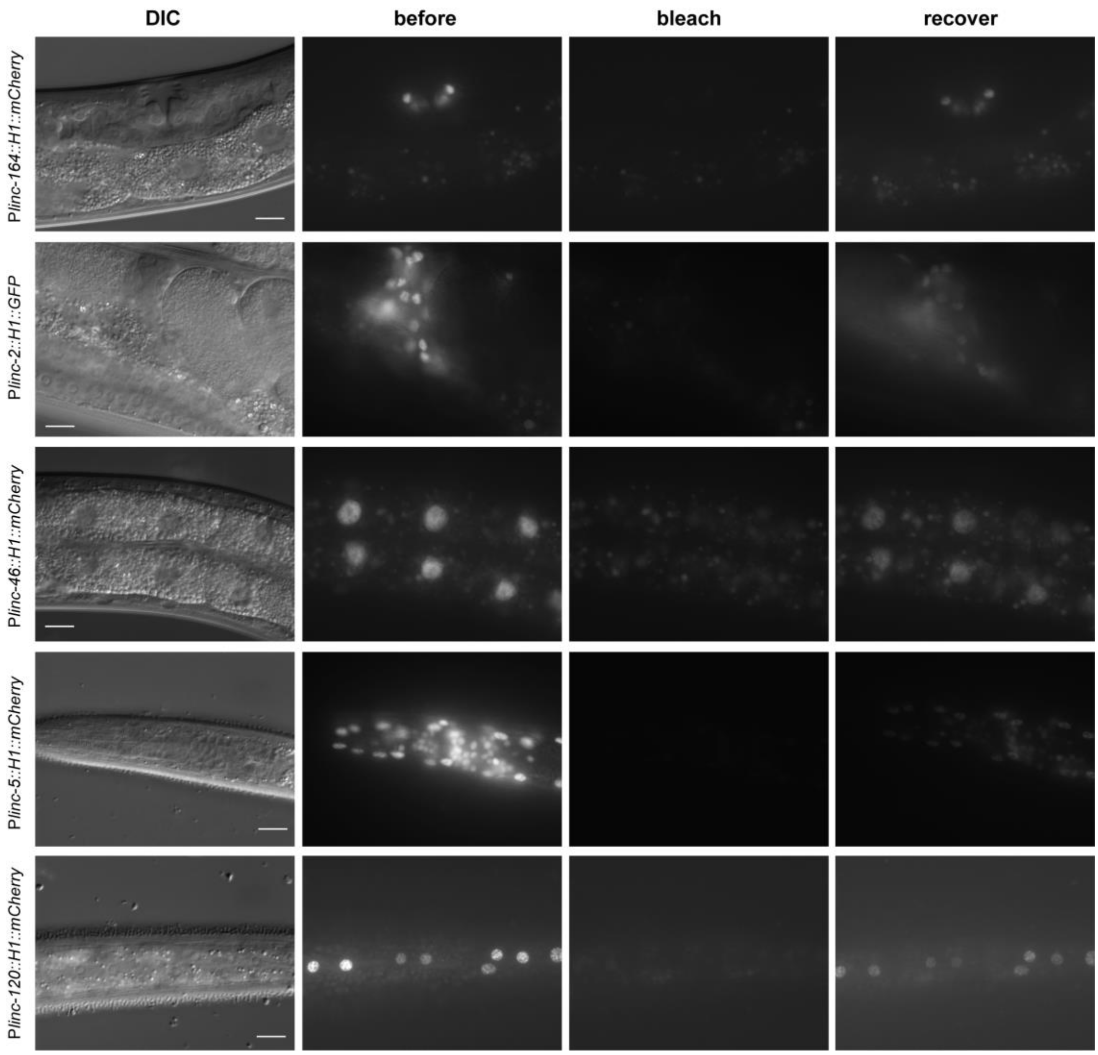


**Supplementary Figure S4. FRAP experiment of five lincRNA reporters.** The exposure time for each PlincRNA::reporter is constant in all photographs. 900 ms for P*linc-164*::*H1*::*mCherry* and P*linc-46*::*H1*::*mCherry*; 150 ms for P*linc-2*::*H1*::*GFP*; 400 ms for P*linc-5*:: *H1*::*mCherry* and P*linc-120*::*H1*::*mCherry*. P*linc-164*::*H1*::*mCherry* and P*linc-46*::*H1*::*mCherry* worms were examined at L4 stage; P*linc-2*::*H1*::*GFP* worms were examined at adult stage; P*linc-5*:: *H1*::*mCherry* and P*linc-120*::*H1*::*mCherry* worms were examined at L2 stage. before = before photobleaching; bleach = immediately after photobleaching; recover = after recovery for 3h at 25°C. Scale bars, 10 μm.

**Description of Supplementary Tables**

Supplementary Table S1. Summary of clone and transgene information of 157 lincRNAs

Supplementary Table S2. Temporal activity of lincRNA expression determined by PlincRNA::reporters and modENCODE RNA-seq

Supplementary Table S3. Single-cell expression data for each PlincRNA::reporter

Supplementary Table S4. Spatial activity of 68 integrated PlincRNA::reporters

Supplementary Table S5 Expression of adjacent protein coding genes for tissue specific lincRNA reporters

Supplementary Table S6. Raw single-cell expression data of the 17 PlincRNA::reporters that have multiple transgenic lines

Supplementary Table S7. Genomic features of the 17 PlincRNA::reporters that have multiple transgenic lines

Supplementary Table S8. Promoter activity of 43 transcription factors in vulva and somatic gonad

Supplementary Table S9. Classification details of cell types

Supplementary Table S10. Explanation of the temporal and spatial activity annotation tables
